# Applying how adults rehearse to understand how rehearsal may develop

**DOI:** 10.3389/fpsyg.2014.01538

**Published:** 2015-01-07

**Authors:** Nelson Cowan, Evie Vergauwe

**Affiliations:** Department of Psychological Sciences, University of MissouriColumbia, MO, USA

**Keywords:** development of rehearsal, verbal rehearsal, short-term memory, working memory, working memory development

We address short-term memory development and the role of covert verbal rehearsal (CVR), the imagined pronunciation of items from a list to be recalled. We suggest insights based on studies of serial and free recall. The research in adults has taken two tacks. (1) Overt rehearsal responses have been elicited to study what CVR strategies may occur, and (2) conditions have been imposed impeding the use CVR, presumably allowing an examination of its effects on recall. There are related lines of developmental research. We evaluate first overt rehearsal and then suppression-of-CVR evidence.

## Overt rehearsal in adults

After Rundus (1971), extensive follow-up research has been conducted (e.g., Tan and Ward, 2008; Bhatarah et al., 2009). Participants produced overt rehearsal between item presentations however they wished, preceding serial or free recall (pre-cued or post-cued). Similar patterns of rehearsal were found in all four situations. Tan and Ward (2008) suggested that “When participants rehearsed, they tended to do so in a cumulative forward order up to Serial Position 4, after which the amount of rehearsal decreased substantially.” After Position 4, assuming that time to rehearse was not the issue, rehearsal could have decreased because of a capacity limit in how many items can be remembered concurrently for rehearsal (Cowan, 2001). Participants appear to want to rehearse from the first item onward and don't find it worthwhile to rehearse subsequences starting later in the list.

One caution, though, is that it is theoretically possible for CVR to occur with words overlapping in time, which is impossible for overt rehearsal. Moreover, findings of Bhatarah et al. (2009) suggest that Tan and Ward's (2008) description is incomplete. Figure 3 of Bhatarah et al. shows the number of words from each input serial position that were overtly rehearsed after Input 1, after Input 2, and so on through Input 8. This figure does show that the frequency of rehearsals decreases as a function of the input position. It also shows, though, a recent effect in rehearsal after each input position, extending to the last three or four items. That pattern could not result from rehearsal always starting at the beginning of the list. Instead, it suggests that sometimes there is rehearsal of recent subsequences of a few items.

This finding of non-initial subsequence rehearsal, not emphasized by Bhatarah et al. (2009), potentially has implications regarding the purpose of CVR. According to Baddeley (1986), the purpose is to reactivate phonological representations before they decay to the point of becoming unavailable for recall. For that purpose, it would make sense to reactivate the least recently encountered or rehearsed items, not the few most recently encountered and not consistently the list from its beginning. Other purposes of rehearsal are possible, however.

Rehearsal could result in a stronger chaining of associations between consecutive words in long-term memory. In that case, one might observe a moving window of rehearsal, e.g., rehearsal of Words 3-4-5, 4-5-6, 5-6-7, etc. We know that chaining cannot be the sole mechanism of recall, given considerable evidence in serial recall against it, favoring item-to-serial-position associations (e.g., Lee and Estes, 1981; Henson et al., 1996). Still, the participant might rehearse a list-medial sequence while thinking about where that sequence is to go in the list, perhaps starting where the previous rehearsal cycle left off.

Another possible purpose of rehearsal is to support grouping, the beginning of the formation of a new chunk in memory (cf., Anderson and Matessa, 1997; Cowan, 2001; Farrell, 2012). If so, a moving window of rehearsal would not be expected. A close analysis might reveal, instead, that participants often rehearse Items 1-2-3 several times and then start rehearsing Items 4-5-6, forming two separate groups, etc. We anticipate in any case that the pattern shown in rehearsal, when viewed in greater detail, will be reproduced in free recall (Laming, 2006).

An objection to the idea of rehearsal in serial recall is that there is not enough time to carry out rehearsal of the list. If the rehearsal following each item does not have to begin at the start of the list, though, there could be enough time, and it could strengthen item-to-item and/or item-to-position associations.

## Development of overt rehearsal

Flavell et al. (1966) exploited the notion that action patterns start out overt and later are internalized, to document the increase in rehearsal for serial recall during childhood. The experimenter pointed to named pictures at a rate of 2 s per picture. In delayed recall, there was a 15-s maintenance period before the child tried to point to the pictures in the presented order. To make children less self-conscious during the retention interval, they wore a space helmet with a translucent visor that came down during the interval but did not cover the mouth. An experimenter trained in lip-reading could see and hear overt verbal rehearsal, mostly in the form of soft whispering or soundless mouth movements. These steadily increased in number from kindergarten (~5 years) to second grade (~7 years) to fifth grade (~10 years), suggesting that rehearsal increases markedly during these elementary school years.

There has been more such work in free recall, providing greater specificity to what is rehearsed. Ornstein and Naus (1978) summarized work showing that, with maturation during the elementary school years, overt rehearsal becomes more cumulative. Guttentag et al. (1987) provided more specificity. They asked children from third through sixth grades (~8 to ~11 years) to think aloud while receiving audiovisual presentations of words for free recall. Overt rehearsal progressed from sets of 1.4 words in third grade to about 4.2 and 4.5 words in fourth and sixth grades. The constraint on rehearsal set size was apparently working memory capacity; all age groups increased the rehearsal set size considerably when each item remained visually available after its presentation (to 2.9, 7.0, and 7.4 in the three age groups). This work has continued with Lehmann and Hasselhorn (2007, 2010, 2012). The 2007 work confirmed directly that capacity limits do restrict the use of cumulative rehearsal, and the later work showed that children's rehearsal patterns conform to adult findings. Sequences in overt rehearsal tended to show up in recall, as in the adult work of Laming (2006), and rehearsal sequences tended toward forward item-to-item associations as in adults (Kahana, 1996). Thus, we suspect that CVR occurs in both serial and free recall and that rehearsal set size increases with age. Rehearsal of several items in a burst often, but not always, begins at list Position 1.

In one study (Cowan et al., 2006a), second-grade children and adults were asked how they carried out a digit span task. Most of the adults vs. none of the second-graders said that they grouped the items, suggesting that there may well be dramatic change in rehearsal style with development.

The natural observation of rehearsal cannot establish the causal role of rehearsal in recall. A positive intervention might, but an attempt to increase the speed of rehearsal to help recall was unsuccessful; the speed of rehearsal could not be increased (Hulme and Muir, 1985). In principle, a causal role of rehearsal might be established by impeding rehearsal and examining the effect on recall, as we now discuss.

## Impeding rehearsal in adults

Short-term memory capacity is diminished when rehearsal is impeded (Baddeley, 1986). To establish a causal connection between CVR and recall, one must take certain precautions. One must ensure that the effect of impeding rehearsal is not due to the interference effects of the rehearsal-suppression task. These interference effects occur when the rehearsal-suppression stimulus changes over time (Oberauer and Lewandowsky, 2008). One also must ensure that the stimulus is encoded phonologically, making it suitable for CVR; suppressing rehearsal impedes phonological encoding for printed but not for spoken stimuli (Baddeley, 1986).

One expectation is that, if CVR is causal, suppression of rehearsal will reduce or eliminate effects attributed to CVR, including detrimental effects of phonological similarity and word length. Cowan et al. (1987) presented lists of words for serial recall that were phonologically similar (*bat, hat*, etc.) or dissimilar, through headphones to ensure phonological encoding, and sometimes required various kinds of rehearsal suppression in a whispered voice during the presentation. The suppression task changed during some trials (e.g., successive letters of the alphabet to be whispered after each list item) and sometimes remained the same (the same letter to be whispered after all list items). Word span was reduced the most overall when the suppression letters changed during the trial, as Oberauer and Lewandowsky (2008) would expect. Even when the suppression item did not change, though, the phonological similarity effect was diminished almost as much as when the suppression items did change within a trial. Thus, beyond interference effects of changing suppression stimuli, we believe that suppression from a repeated stimulus hinders recall by preventing phonological rehearsal during presentation of the list.

Instead of relying on simple quantitative effects of suppressing rehearsal, one can set up tasks allowing alternative strategies that either include or exclude CVR. Cowan et al. (2006b) set up a task in which names appeared one at a time at different house locations. The participant received a name probe and was to drag it to the house in which it had appeared. Sometimes the names had to appear one to a house, and a simple strategy was to remember separately the path between houses and the serial sequence of names. For example, the third name in the remembered list must have appeared in the third house in the remembered path. On other trials, some houses remained vacant but multiple names appeared at other houses (not in immediate succession), so that the path between houses became convoluted. Then, remembering the serial sequences of houses was difficult and the participant had to rely on direct house-name associations. The results showed that adults tended to use the path- and word-sequencing method (doing better when the paths were not convoluted) unless rehearsal suppression was required, in which case they switched to the house-name association method (doing better when the paths were convoluted because fewer houses had names). It was suggested that the path and sequence-rehearsal method required less attention.

## Impeding rehearsal: a comparison with children

One might attempt to impede CVR in children. If young children do not rehearse effectively, impeding rehearsal should have little effect on their recall. In practice, this research strategy is problematic because rehearsal is more attention-demanding in young children than it is in adults (Guttentag, 1984).

Another research strategy is to ask whether suppression of rehearsal in adults makes them perform like children. Cowan et al. (1987) did find that rehearsal suppression made adults resemble 6- to 8-year-old children in their level of recall performance and magnitude of the phonological similarity effect; caution is needed in interpreting age changes in the magnitude of the phonological similarity effect, however (Jarrold and Citroën, 2013). Cowan et al. (2006b) found that third-grade children showed a pattern of recall suggesting a direct word-house association method that did not require CVR, much like adults under rehearsal suppression.

## Conclusion

In traditional approaches toward rehearsal, it is needed to reactivate items in a repeating loop before they decay. There are several reasons to suspect that this function of rehearsal is discrepant from the overt rehearsal protocols and recall records, and therefore is not suitable to explain the development of rehearsal. We have suggested some alternative schedules and functions of rehearsal (a moving window to strengthen associations, or separate item groups), and have presented evidence suggesting that rehearsal increases with development in a manner that might assist both serial and free recall. Capacity limits may restrict young children's ability to use rehearsal, in which case rehearsal can be said to amplify the benefits of the growth of capacity. It is possible that gradual, quantitative changes in rehearsal ability have abrupt, qualitative, step-like consequences on the development of recall because a certain level of ability may be needed to make CVR actually useful in a given recall task.

### Conflict of interest statement

The authors declare that the research was conducted in the absence of any commercial or financial relationships that could be construed as a potential conflict of interest.
